# Renin–Angiotensin System Activation Drives Early Cardiorenal Dysfunction in Normotensive Prediabetes

**DOI:** 10.1002/edm2.70363

**Published:** 2026-10-19

**Authors:** Nour‐Mounira Z. Bakkar, Rana Ghazi, Nahed Mougharbil, Safaa H. Hammoud, Ramzi Sabra, Ali H. Eid, Ahmed F. El‐Yazbi

**Affiliations:** ^1^ Department of Pharmacology and Toxicology, Faculty of Medicine American University of Beirut Beirut Lebanon; ^2^ Department of Pharmacology and Therapeutics Beirut Arab University Beirut Lebanon; ^3^ Department of Basic Medical Sciences, College of Medicine, QU Health Qatar University Doha Qatar; ^4^ Research and Innovation Hub Alamein International University Alamein Egypt; ^5^ Department of Pharmacology and Toxicology, Faculty of Pharmacy Alexandria University Alexandria Egypt; ^6^ Department of Chemistry and Biochemistry Texas Tech University Lubbock Texas USA

**Keywords:** baroreceptor sensitivity, cerebrovascular active tone, prediabetes, renal perfusion, renin‐angiotensin‐system (RAS)

## Abstract

**Introduction:**

Prediabetes is increasingly recognised as a stage at which cardiovascular and renal dysfunction begin to develop despite the absence of overt metabolic disease. The mechanisms underlying these early alterations remain poorly understood. Because adipose tissues surrounding blood vessels and kidneys can locally generate components of the renin–angiotensin system (RAS), we hypothesised that subclinical RAS activation contributes to early cardiorenal dysfunction in prediabetes.

**Methods:**

Using a pre‐established normotensive, non‐obese rat model of prediabetes induced by 12 weeks of mild hypercaloric feeding, we examined the impact of systemic angiotensin II (AngII) elevation and angiotensin‐converting enzyme (ACE) inhibition on cardio‐, cerebro‐ and renovascular function by invasive hemodynamics, ex vivo pressure myography, and ex vivo renal perfusion experiments, respectively.

**Results:**

Prediabetic rats exhibited impaired parasympathetic baroreceptor sensitivity and altered renovascular endothelium‐dependent vasodilation despite preserved blood pressure and cardiac function. Systemic AngII infusion further exacerbated autonomic and renovascular dysfunction. In contrast, treatment with a non‐hypotensive dose of the ACE inhibitor captopril restored baroreceptor sensitivity, improved renovascular endothelial function, reduced brainstem inflammation, and attenuated renal tubular fibrosis. Examination of perivascular and perirenal adipose tissues revealed significantly higher protein concentration of ACE1 in prediabetic rats compared to controls.

**Conclusion:**

These findings are consistent with an early activation of RAS contributing to subclinical cardiovascular and renal dysfunction during prediabetes independently of hypertension. Targeting RAS signalling at this early stage may therefore represent a strategy to limit progression of cardiorenal complications along the continuum from prediabetes to diabetes.

## Graphical abstract

Increased angiotensin‐converting enzyme 1 (ACE1) expression in perivascular and perirenal adipose tissues of prediabetic rats contributes to early cardiovascular and cardiorenal deterioration in the absence of obesity and hypertension. Systemic Angiotensin II exacerbates the observed detrimental effects of prediabetes. Treatment with non‐hypotensive captopril salvages early prediabetic cardiorenal aberrations.

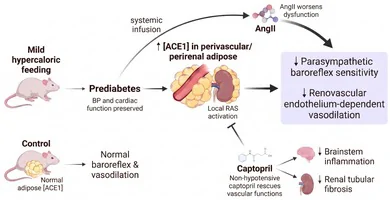

## 1 Introduction

Diabetes is a known risk factor for the development of cardiovascular and renal dysfunction [1, 2]. In fact, cardiovascular disease is the primary cause of death in diabetic patients. Diabetes is often confounded with hypertension, coronary heart disease, atherosclerosis, and congestive heart failure, all of which encompass a considerable inflammatory component.

The renin angiotensin system (RAS), a humoral orchestrator of homeostasis via the modulation of blood pressure (BP) and a chief driver of hypertension, contributes to diabetic cardiovascular complications [3]. The RAS operates through two counter‐acting arms: angiotensin‐converting enzyme 1‐Angiotensin II‐Angiotensin II receptor 1 (ACE1‐AngII‐AT1R) and angiotensin‐converting enzyme 2‐Angiotensin(1–7)‐Mas receptor (ACE2‐Ang(1–7)‐MasR) [4]. Overactivation of the ACE‐AngII‐AT1 axis is associated with detrimental effects like vascular remodelling and elevated cardiac afterload. Accordingly, hypertension is managed by blocking either AngII formation, through inhibiting ACE, or AngII signalling along AT1 receptors.

Type 2 diabetes (T2D) is preceded by a prediabetic stage associated with significant metabolic impairment and cardiovascular involvement, providing a window for early intervention [5, 6, 7, 8]. In fact, the igniting factor of diabetic cardiovascular events is believed to be triggered by early adipose tissue dysfunction, prior to the establishment of hyperglycemia [9, 10]. Obesity is a significant risk factor for the development of prediabetes. According to the Framingham Heart Study, excess weight is significantly associated with essential hypertension risk, with adiposity being a significant, independent predictor of hypertension [11, 12, 13]. Importantly, beyond systemic AngII levels, local tissue production of AngII conduces vascular stiffness and injury [14, 15, 16].

Adipose tissues possess depot‐specific characteristics rendering their metabolic response to fat accumulation with chronic hypercaloric intake differential [17, 18, 19]. Hypertrophic growth of adipose tissues, as opposed to hyperplastic expansion, which involves an increase in the number of adipocytes, promotes hypoxia‐driven pro‐inflammatory pathways culminating in and propelling cardiovascular deteriorative processes [20]. Adipose pads enveloping the heart, blood vessels, and kidneys possess the capacity to adopt a beige phenotype under specific stimuli rendering them metabolically distinct. Owing to their anatomical proximity to the cardiorenal system, pathological changes in epicardial, perivascular (PVAT), and perirenal adipose tissues (PRAT) significantly alter the function of underlying organs through paracrine and/or systemic spillage of pro‐inflammatory adipocytokines [21, 22, 23]. Hence, intervening at the level of the mechanisms instigating adipose tissue dysfunction offers the opportunity of interrupting cardiovascular detrimental mechanisms [18, 20, 24].

Previous studies have demonstrated that multiple components of the RAS exist in adipose tissues, contributing to local and systemic AngII concentrations [25]. Adipose tissue levels of AngII are elevated in obesity and/or T2D and contribute to adipose inflammation in a vicious cycle [26, 27]. Hence, it is plausible that interrupting AngII production at the level of PVAT and PRAT can mitigate, ameliorate, or delay prediabetic cardiovascular detrimental processes. Research shows an established role of elevated systemic AngII levels in hypertension and its impact on the cardiovascular system; however, much less is known about subclinical RAS activation in inflamed adipose depots and its contribution to the propagation of cardiovascular disease in prediabetes.

We have previously established a rat model of prediabetes based on 12 weeks of mild hypercaloric (HC) feeding [28]. This prediabetic rat demonstrates early signs of cardiovascular deterioration in the absence of obesity and hypertension allowing for the characterisation of the early insidious deteriorative processes culminating in cardiovascular disease. Molecular profiling of the mild‐hypercaloric‐fed rat provided evidence of localised PVAT and PRAT inflammation conducing prediabetic cardiovascular and renovascular dysfunction, as well as cardiac autonomic neuropathy, which are responsive to treatment with non‐hypoglycemic doses of anti‐diabetic drugs with anti‐inflammatory effects, such as metformin and pioglitazone, or dietary interventions like phosphorus supplementation or therapeutic fasting [29, 30, 31, 32].

In this study, we explore and characterise the potential contribution of subclinical RAS activation to the observed cardiovascular and renal detrimental processes through treatment of HC‐fed, prediabetic rats with a low, non‐hypotensive dose of the ACE inhibitor, captopril. We employ an AngII pump to isolate the impact of elevated systemic versus localised AngII levels.

## 2 Materials and Methods

### 2.1 Ethical Approval

All animal experiments were carried out in compliance with the study protocol number 16–10‐386 approved by the AUB Institutional Animal Care and Use Committee (IACUC) in accordance with the Guide for the Care and Use of Laboratory Animals of the Institute for Laboratory Animal Research of the National Academy of Sciences, U.S.A.

### 2.2 Computational Analysis

Computational assessment of the role of ACE signalling in the pathological pathways involved in prediabetes, cardiac autonomic neuropathy, and nephropathy was performed as described in our previous work [33]. Briefly, the gene sets for protein targets implicated in prediabetes, cardiac autonomic neuropathy, nephropathy, and RAS signalling in humans were downloaded from the STRING Consortium website at https://string‐db.org/. Afterwards, the GeneVenn Tool (https://www.bioinformatics.org/gvenn/index.htm) was used to map the overlap among the proteins in different pathways. STRING program (Ver. 11.5/12.0, https://cn.string‐db.org) was then used to generate protein–protein interaction networks among the common proteins and assess the statistical significance of the protein–protein interaction.

### 2.3 Experimental Design

We utilised a pre‐established rat model of prediabetes based on chronic, mild HC feeding [28, 29, 31, 32, 34, 35]. Details for model reproduction are available in a published protocol [28]. Briefly, 4–5‐week‐old male Sprague Dawley rats (average weight = 150 g) were fed a mild HC diet providing 4.035 kcal/g as opposed to normal chow (NC, ENVIGO, Teklad Rodent Diets, Madison, WI) with 3 kcal/g for 12 weeks. HC diet was prepared in house by addition of 20% by weight food‐grade fructose (Santiveri Foods, Spain) and 15% by weight hydrogenated vegetable oil (Mazola, BFSA) and supplementation of major electrolytes and vitamins to match ENVIGO diet composition and comply with recommendations of the American Institute of Nutrition. Hence, the HC diet constituted 46.13% w/w carbohydrates, 15.8% w/w protein, and 18.06% w/w fat. The final calorie content for NC vs. HC diets, respectively, was distributed as follows: 54% vs. 45.73% carbohydrates, 32% vs. 15.66% protein, and 14% vs. 38.68% fat (of which 0.9% vs. 5% by weight saturated), respectively. Previous metabolic profiling of the mild HC‐fed rat model consistently revealed hyperinsulinemia as well as dyslipidemia in the form of non‐HDL hypercholesterolemia and hypertriglyceridemia after 12 weeks of feeding [30, 31, 34, 35]. HC‐fed rats develop hyperglycemia, assessed as random and fasting blood glucose levels, following 24 weeks of feeding [31].

Rats were randomly divided into 6 groups (*n* = 8/group), all of which were fed respective diets for 12 weeks and underwent subcutaneous, mid‐scapular mini osmotic pump (AlzetMODEL 2ML2) implantation for continuous infusion of either saline or AngII at week 10: (1) Saline‐infused (sham) control rats fed normal chow (*Control*), (2) AngII‐infused rats fed normal chow (*Control‐Ang*), (3) saline‐infused rats fed normal chow and treated with 10 mg/kg of captopril daily starting week 9 (*Control‐CAP*), (4) saline‐infused HC‐fed rats (*HC*), (5) AngII‐infused HC‐fed rats (*HC‐Ang*), and (6) saline‐infused HC‐fed rats treated with 10 mg/kg captopril daily starting week 9 (*HC‐CAP*). 0.8 mg/kg/day AngII was infused at a rate of 5 μL/h for 14 days. Captopril treatment was freshly prepared daily by crushing a 25 mg tablet, suspending it in water, and dissolving the required volume in a 5 g wet‐chow pellet. Rats were monitored to ensure that the pellet was completely consumed before returning to ad libitum feeding. The selected doses of AngII and CAP were determined in preliminary experiments to be devoid of gross hemodynamic responses in control rats within the time window of treatment.

### 2.4 Calorie Intake, Body Weight and Body Composition Measurements

Daily calorie intake was measured based on mass of food consumed multiplied by the calorie content per gram of the corresponding diet. Body weight was measured weekly along with the anus‐to‐nose length. Fat‐to‐lean (fat:lean) ratio was calculated according to fat and lean densities acquired using a nuclear magnetic resonance machine (LF10 Minispec NF4433, Bruker, MA, USA). The values for body weight and fat:lean ratio reported here were recorded before sacrifice, that is, at week 12.

### 2.5 Fasting Blood Glucose and Glycated Haemoglobin Levels

Rats were fasted overnight before sacrifice. On the day of the sacrifice, fasting blood glucose concentration was measured via Accu‐check Performa glucometer (Roche Diagnostics, Basel, Switzerland) and HbA1C level was obtained via a bench‐top Labona Check A1C analyser (Greencross Medis, Cheonan‐Si, South Korea) using blood drops collected from the tail vein.

### 2.6 Non‐Invasive Blood Pressure and Echocardiography

Non‐invasive BP was measured at week 12 by a tail cuff using the CODA High Throughput Monitor (Kent Scientific, Torrington, CT) which is based on a volume pressure recording sensor, as previously described [29, 35, 36]. Prior to BP measurement, rats were sedated using 70% of a 1:4 v/v mixture of 1.034 mg/mL/kg ketamine/xylazine intraperitoneal injection. Echocardiography was then performed on sedated rats along the parasternal long‐axis B‐ and M‐modes using SonixTouch Q+ ultrasound (BK ultrasound, Peabody, MA) to assess cardiac function, namely, ejection fraction and fractional shortening [36].

### 2.7 Invasive Hemodynamic Study and Baroreceptor Sensitivity Assessment

To assess baroreceptor sensitivity (BRS), a previously established and described invasive hemodynamic workup protocol was conducted [29, 32, 35, 36]. Briefly, rats were lightly anaesthetised using 100 mg/mL thiopental (AUBMC Pharmacy) before tracheostomy was performed. The carotid artery and the jugular vein were then isolated and instrumented with 8‐gauge polyethylene catheters. The catheter inserted in the carotid artery was connected to a Millar pressure transducer, while that in the jugular vein was connected to a shunt for IV injection of vasoactive drugs. Rats were allowed a 30‐min stabilisation period prior to drug administration. Increasing bolus doses of phenylephrine (PE, 0.25, 0.5, 0.75, 1, and 2 μg) and sodium nitroprusside (SNP, 0.5, 1, 2, 4, and 8 μg) were administered at roughly 5‐min intervals, allowing enough time for baseline MAP to be restored between doses. PowerLab hardware and LabChart Pro 8 software (AD Instruments Ltd., Dunedin, New Zealand) were utilised for data acquisition. BRS was defined as the slope of linear regression of the change in heart rate (ΔHR) to the change in mean arterial pressure (ΔMAP).

### 2.8 Ex Vivo Pressure Myography

To assess cerebrovascular tone, pressure myography was conducted as previously described [34, 37]. Pressure ramp experiments were performed on rat middle cerebral arteriole segments as follows: after the establishment of myogenic tone, the pressure was dropped to 10 mmHg and then gradually increased in a stepwise manner to 20, 40, 60, 80, 100, 120, and 140 mmHg. The outer vessel diameter was measured at every step. The arteriolar segment was then washed, the pressure dropped back to 10 mmHg, and the same experiment was repeated with arteriolar segments immersed in calcium‐free buffer (pH 7.4) containing 130 mM NaCl, 4 mM KCl, 1.2 mM MgSO_4_.7H_2_O, 4 mM NaHCO_3_, 10 mM HEPES, 1.18 mM KH_2_PO_4_, 6 mM Glucose, 0.03 mM EDTA, and 2 mM EGTA. Active tone was calculated as the difference of outer arteriole diameters in calcium‐containing and calcium‐free buffer at the corresponding pressure points.

### 2.9 Ex Vivo Renal Perfusion Assessment

Renovascular function was evaluated using the ex vivo kidney perfusion experiment, as previously described [35]. Briefly, the left kidney was isolated, and its renal artery was ligated and catheterised using an 18‐gauge needle. The kidney was then flushed with heparinised saline and perfused at 37°C with a constant flow rate of Krebs solution delivered via a peristaltic pump (Model P3; Pharmacia Fine Chemicals, Sweden). Krebs solution was composed of 120 mM NaCl, 5 mM KCl, 2.5 mM CaCl_2_, 1.2 mM MgSO_4_ · 7H_2_O, 1.2 mM KH_2_PO_4_, 25 mM NaHCO_3_ and 11 mM glucose.

Renal vascular tone was established via continuous infusion of the α_1_‐adrenergic agonist, phenylephrine (PE). To assess endothelium‐dependent vasodilation, increasing doses of carbachol (CCh, 1 × 10^−12^ to 1 × 10^−5^ M) were added to the Krebs solution. The status of nitric oxide‐ and prostaglandin‐mediated vasodilation was explored using nitric oxide synthase inhibitor, N_ω_‐nitro‐L‐arginine (L‐NAME, 100 μM), and non‐selective cyclooxygenase inhibitor, diclofenac (7 μM), respectively, injected into the perfusion solution.

### 2.10 Sacrifice and Organ Harvest

Anaesthetised rats were sacrificed by decapitation. Organs were collected and either snap‐frozen in liquid nitrogen and then stored at −80°C for molecular probing, or placed in 10% formaldehyde for histological analysis.

### 2.11 Histopathological and Immunohistochemical Examination

Formaldehyde‐preserved thoracic aortas and right kidneys were fixed in 100% ethanol, paraffin embedded, cut transversely, and mounted on slides. Haematoxylin and eosin (H&E) stain was utilised to examine medial thickness of thoracic aortas. Periodic Acid‐Schiff stain was used to assess glomerular structure as the ratio of the mesangial capsule to the glomerular area, while Masson trichrome was employed for tubular fibrosis examination [30, 32].

IBA‐1 immunostaining was performed on frozen brainstem sections as a marker of microglia. Briefly, frozen sections were mounted on slides, dewaxed by heating at 55°C for 30 min, then progressively hydrated using alcoholic solutions of increasing water content (xylol 2%, xylol 1%, ethanol 100%, ethanol 95%, ethanol 75%, water). Slides were then incubated in antigen retrieval buffer made of 10 mM sodium citrate and heated in a steamer for 15 min. Upon cooling and washing, blocking buffer of 10% normal goat serum, 1% bovine serum albumin, and tris‐buffered saline (TBS) was added and kept for two hours before washing and adding rabbit anti‐IBA‐1 (1:100, abcam). Following overnight incubation at 4°C, the slides were washed with TBS‐triton twice and 0.3% H_2_O_2_ was added for 15 min to quench internal peroxidases. Slides were then incubated for one hour in horseradish peroxidase (HRP) and washed before the addition of DAB for 10 min. Haematoxylin counterstaining was performed prior to microscopic examination.

### 2.12 Enzyme‐Linked Immunosorbent Assay (ELISA)

Protein concentration of rat angiotensin I converting enzyme (ACE) in PVAT and PRAT was measured using ELK Biotechnology ELISA kit (Cat. ELK 1398) as per the manufacturer's instructions. Briefly, tissues (*n* = 3/condition) were lysed on ice using PBS (w:v = 1:9), tissue homogenates were centrifuged at 1000× *g*, and the supernatant was collected and assayed.

### 2.13 Statistical Analysis

Data is presented as mean ± SEM. Statistical significance was tested using either student *t*‐test (for comparison of two groups, i.e., Control vs. HC), one‐way analysis of variance (ANOVA, for comparison of more than two groups) followed by Tukey post hoc test, or two‐way repeated measures ANOVA followed by Sidak's post hoc test for comparisons between groups across different doses of phenylephrine or sodium nitroprusside in hemodynamics, pressure levels in pressure myography, or concentrations of carbachol in renal perfusion experiments, as indicated in the figure legends of the corresponding experiment, using GraphPad Prism version 7 (San Diego, CA). *p* < 0.05 was considered statistically significant. Effect sizes are reported as Cohen's *d* for *t*‐tests, eta squared (*η* ^2^) for one‐way ANOVA, and partial eta squared (*η*_*p*_^2^) for the interaction between the rat groups and the pressure/concentration effect in two‐way repeated measures ANOVA. For *t*‐tests, 95% confidence interval (CI) for the difference between two groups are reported.

## 3 Results

### 3.1 Computational Assessment of the Role of ACE Signalling in Prediabetes and the Associated Cardiorenal Complications

In addition to overlap, protein–protein interaction networks showed significant interactions among the protein targets implicated in prediabetes, nephropathy, autonomic neuropathy together with those involved in the renin‐angiotensin system (Figure 1A, *p* < 1 × 10^−16^). The protein network shows that both ACE and AngII are central to the interaction pathways within the three disorders. Upon mapping the overlap among prediabetes, nephropathy, and autonomic neuropathy, the three disorders appeared to converge on abnormal insulin signalling (Figure 1B). Further examination of the potential interactions among insulin and the protein targets involved in the renin‐angiotensin signalling also revealed significant interactions (*p* < 1 × 10^−16^) between insulin and various targets including ACE, AngII, renin, and AT1 receptor (Figure 1C).

**FIGURE 1:**
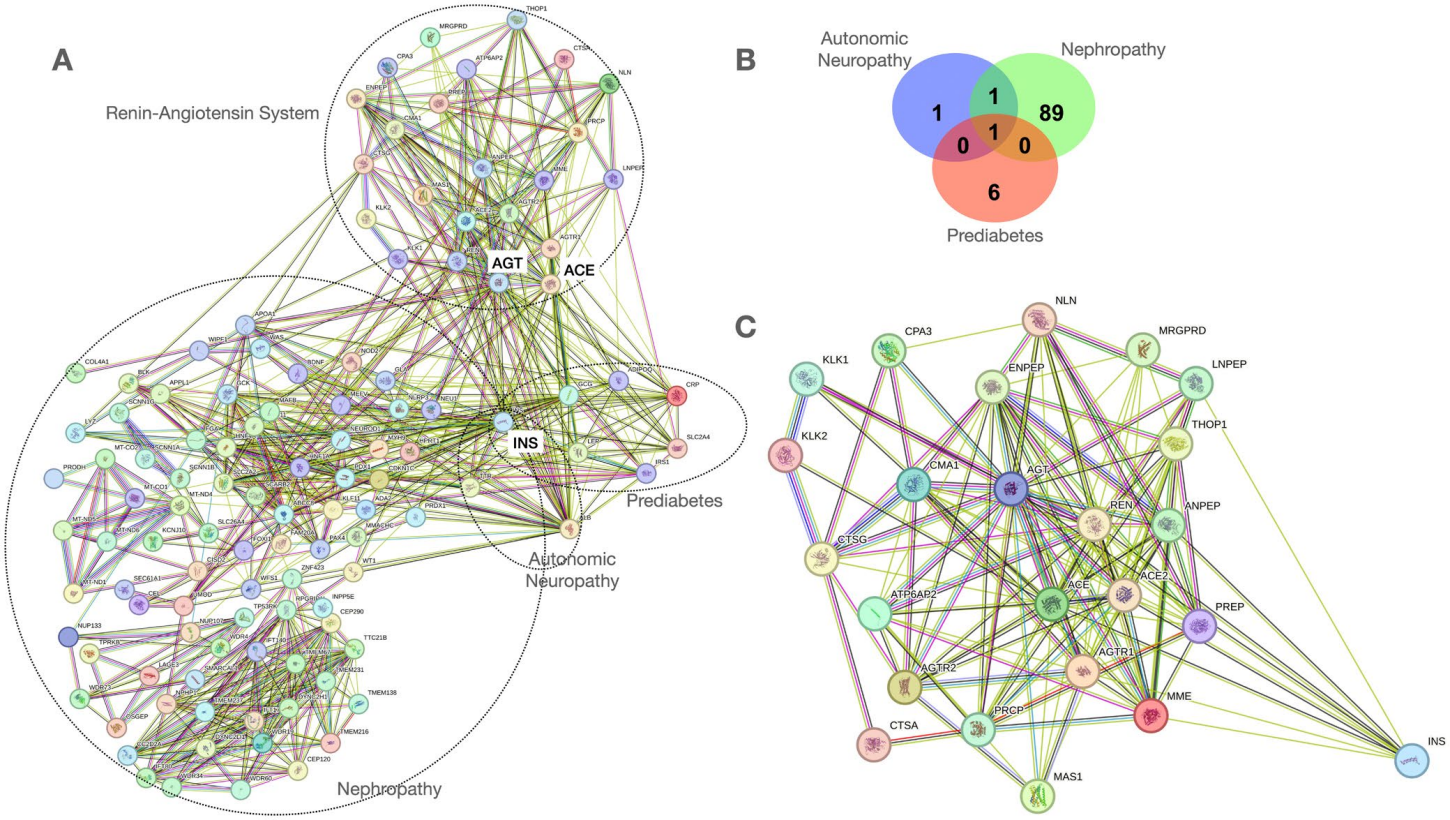
Computational assessment of the interplay among protein targets involved in the renin–angiotensin system and those implicated in the pathology of prediabetes and its complications of nephropathy and autonomic neuropathy. (A) Protein–protein interaction network of all protein targets in the four categories; (B) Overlap in protein targets among prediabetes and its complications; (C) Protein–protein interaction network among insulin and the proteins in the renin–angiotensin system. ACE, angiotensin‐converting enzyme; AGT, angiotensin‐II; INS, insulin.

### 3.2 Metabolic State and Glycemic Control in the Prediabetic Rat

From week 1 through 12, rats fed an HC diet consumed significantly higher calories than their control, normal chow‐fed counterparts (Figure 2A). HC rats retained a comparable weight to that of the control group at 12 weeks (Figure 2B). However, HC rats with AngII pumps demonstrated a drop in body weight with respect to their HC counterparts. Despite the absence of obesity, that is, elevated body weight, in response to chronic HC feeding, HC‐fed rats exhibited significantly higher fat‐to‐lean ratio compared to controls, normal‐chow‐fed rats (Cohen's *d* = 1.39, 95% CI = 0.11–0.57). Rats of all groups had similar fasting blood glucose levels (BGL) (Figure 2C) and glycated haemoglobin (HbA1C) levels (Figure 2D) indicating the absence of diabetes.

**FIGURE 2:**
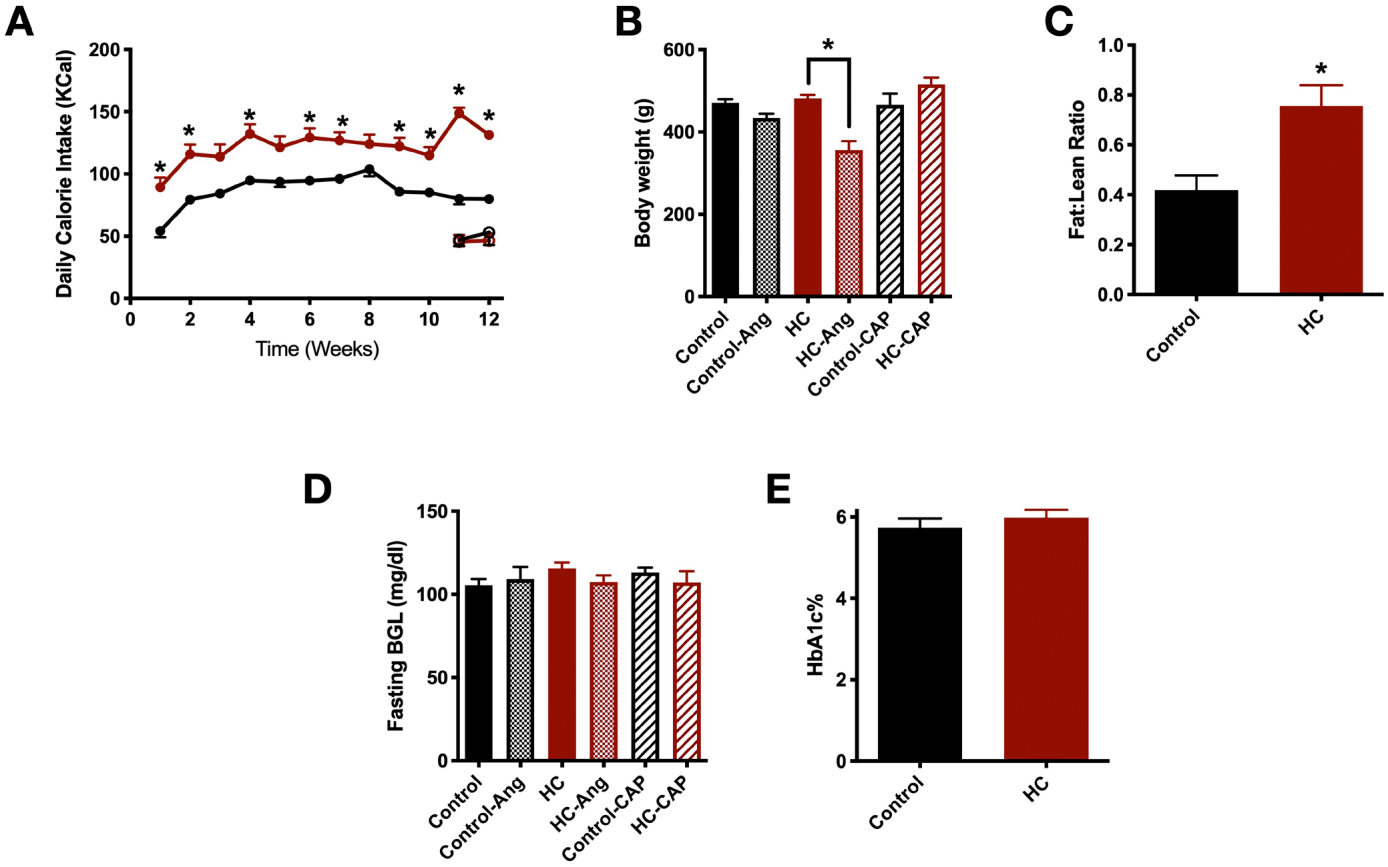
Daily calorie intake, metabolic, and glycemic manifestations of high calorie (HC) feeding. (A) Daily calorie intake (KCal) of control vs. HC‐fed rats for 12 weeks, (B) Body weight (g) measured at week 12 before sacrifice, (C) Fat:lean ratio, in control and HC‐fed rats, calculated using fat and lean tissue densities obtained via nuclear magnetic resonance at week 12, (D) Fasting blood glucose levels (BGL, mg/dL) measured using tail vein blood drop at week 12 after an overnight fast, (E) Glycated haemoglobin (HbA1c%) at week 12 for control and HC‐fed rats. (Control: *n* = 8, HC: *n* = 8, Control‐Ang: *n* = 7, HC‐Ang: *n* = 8, Control‐CAP: *n* = 7, HC‐CAP: *n* = 7). Values are presented as mean ± SEM. Statistical significance was determined using unpaired student *t*‐test for A, C, and E and one‐way ANOVA followed by Tukey post hoc test for B and D. * denotes *p* < 0.05.

### 3.3 Cardiovascular Function

HC‐fed rats had similar non‐invasive systolic BP levels (Figure 3A), ejection fraction (Figure 3B), and fractional shortening (Figure 3C) compared to controls, confirming the absence of decompensated, overt cardiovascular disease in the employed prediabetic rat model. Interestingly, chronic systemic AngII infusion resulted in a significant elevation in systolic BP in HC‐fed rats only (*η* ^2^ = 0.32) (Figure 3A), reflecting a putative compounding effect of systemic AngII on co‐existing, metabolically induced, localised AngII levels driving hypertension. Treatment of control and HC‐fed rats for two weeks with the ACE inhibitor, Captopril, had no effect on BP at week 12 (Figure 3A), ruling out a role for the hypotensive effect in further changes seen with the treatment.

**FIGURE 3:**
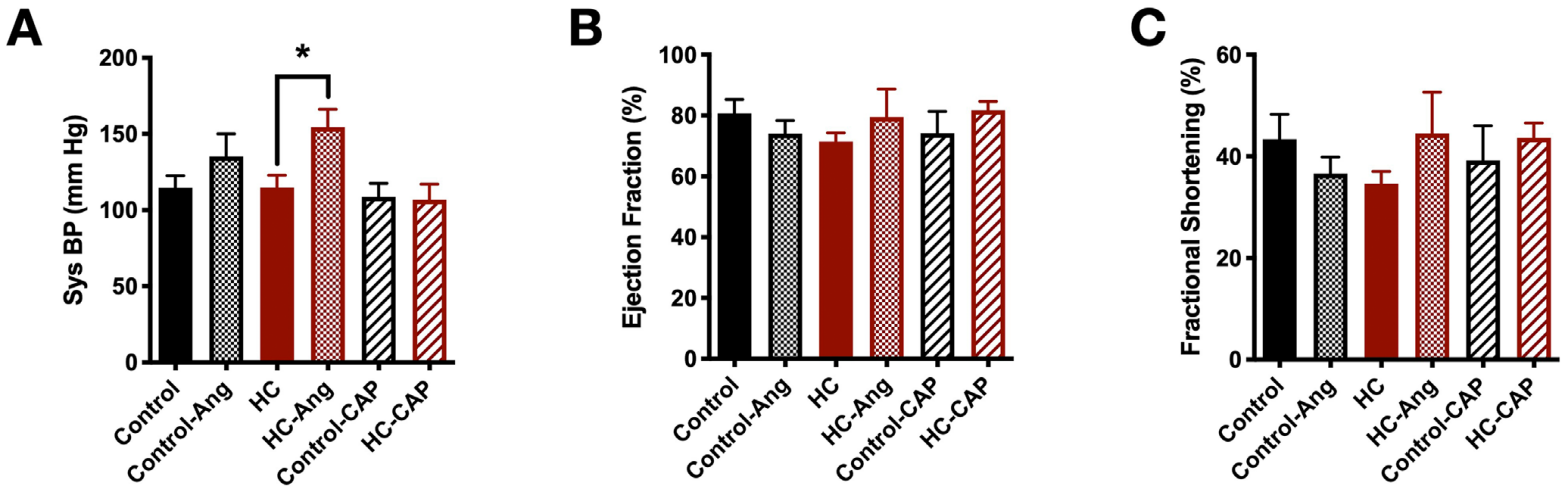
Non‐invasive blood pressure and cardiac function. (A) Systolic blood pressure (mmHg) obtained using CODA tail cuff, (B) ejection fraction (%) and (C) fractional shortening (%) assessed using echocardiography performed at prior to sacrifice at week 12. Statistical significance was determined using one‐way ANOVA followed by Tukey post hoc test. * denotes *p* < 0.05.

### 3.4 Invasive Hemodynamics and Baroreceptor Sensitivity

Invasive hemodynamic assessment revealed subclinical cardiovascular deterioration (Figures 4 and 5). Both NC‐ and HC‐fed rats with AngII infusion mounted an exaggerated pressor response to 2 μg of phenylephrine (PE) demonstrated by a significantly higher mean change in mean arterial pressure (ΔMAP) compared to Control rats (normal‐chow fed) (Figure 5A). However, no significant differences in reflex bradycardic responses to increased BP, assessed as the mean change (i.e., difference) in heart rate (ΔHR) in response to increasing doses of PE, was observed among the rat groups (Figure 5B). However, control rats with AngII infusion pumps and HC‐fed rats (with or without AngII pumps) had blunted parasympathetic baroreceptor sensitivity (BRS) as revealed by significantly shallower slopes of the linear regression of ΔHR versus ΔMAP compared to the control group (Figure 5C,D). In keeping with the potentiating effect of HC feeding on AngII levels, HC‐fed rats with AngII infusion pumps revealed further deterioration of parasympathetic BRS as indicated by a significantly lower slope compared to their HC‐fed counterparts. Treatment of HC‐fed rats with ACE inhibitor, Captopril, ameliorated parasympathetic BRS as evidenced by a significantly higher slope compared to untreated HC‐fed rats (*η* ^2^ = 0.68).

**FIGURE 4:**
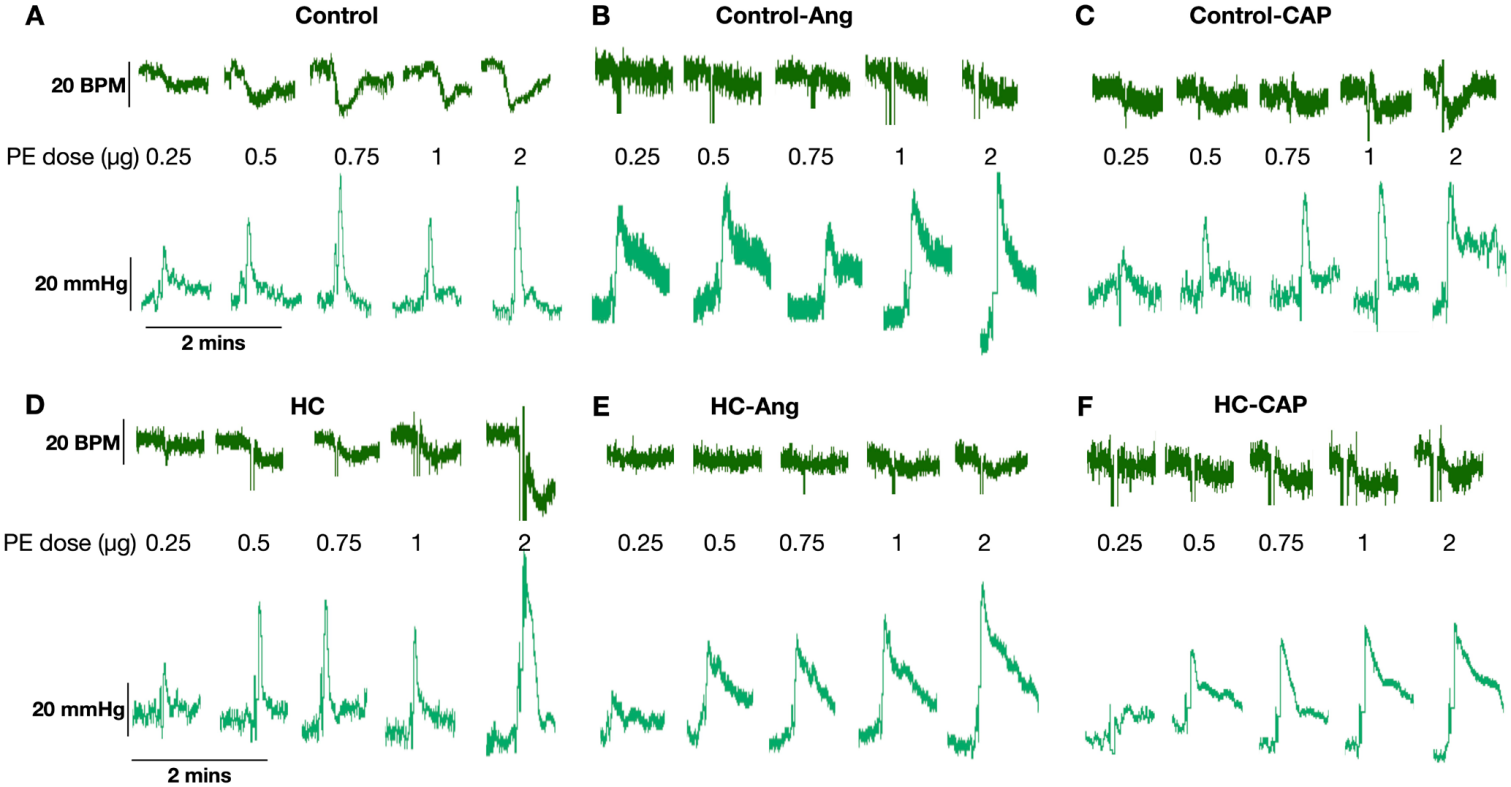
Representative tracings of parasympathetic responses to phenylephrine. Reflex bradycardic responses to blood pressure increments at increasing doses of the vasopressor, phenylephrine (PE) in (A) Control, (B) Control‐Ang, (C) Control‐CAP, (D) HC, (E) HC‐Ang, and (F) HC‐CAP rats.

**FIGURE 5:**
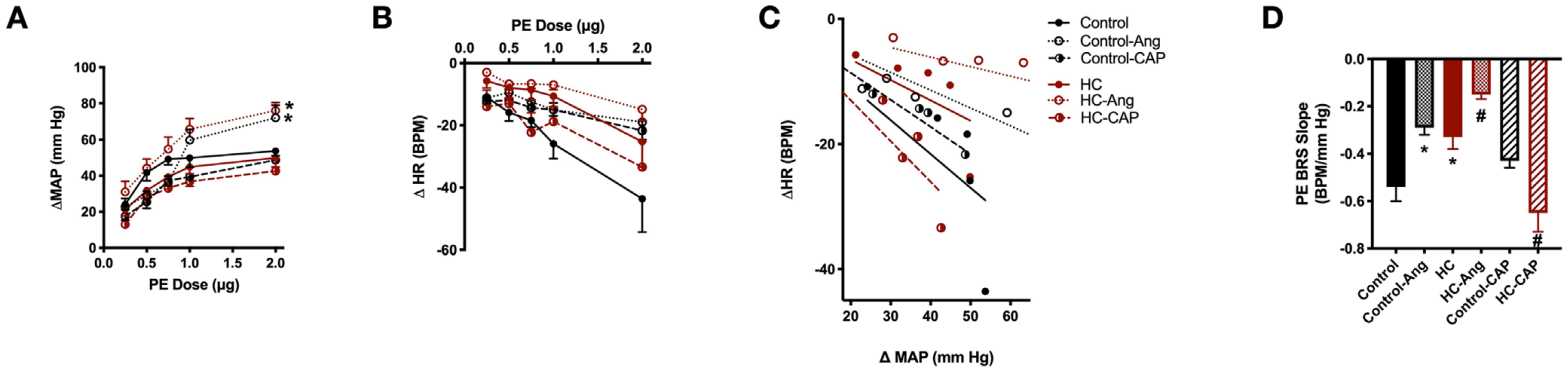
Parasympathetic baroreceptor sensitivity assessed by invasive hemodynamic workup. (A) Changes in mean arterial pressure (ΔMAP, mmHg) and (B) Changes in heart rate (ΔHR, BPM) in response to increasing doses of phenylephrine (PE), (C) linear regression of ΔHR vs. ΔMAP, and (D) parasympathetic baroreceptor sensitivity, as the slope of the regression line in the different rat groups. (Control: *n* = 8, HC: *n* = 8, Control‐Ang: *n* = 7, HC‐Ang: *n* = 8, Control‐CAP: *n* = 7, HC‐CAP: *n* = 7). Values are presented as mean ± SEM. Statistical significance was determined by one‐way ANOVA followed by Tukey's post hoc test for the comparison of baroreceptor sensitivity slopes and two‐way repeated measures ANOVA followed by Sidak's post hoc test for the dose‐effect curves (A & B). * and # denote *p < 0.05* versus control and HC rats, respectively.

Consistent with the results of previous studies in our laboratory, depressor responses to sodium nitroprusside (SNP) (Figures 6 and 7A), reflex tachycardic responses to drops in BP (Figures 6 and 7B), and sympathetic BRS (Figures 6 and 7C,D) were intact in the prediabetic, HC‐fed rat. AngII infusion had no effect on depressor responses to SNP, tachycardic responses, and sympathetic BRS.

**FIGURE 6:**
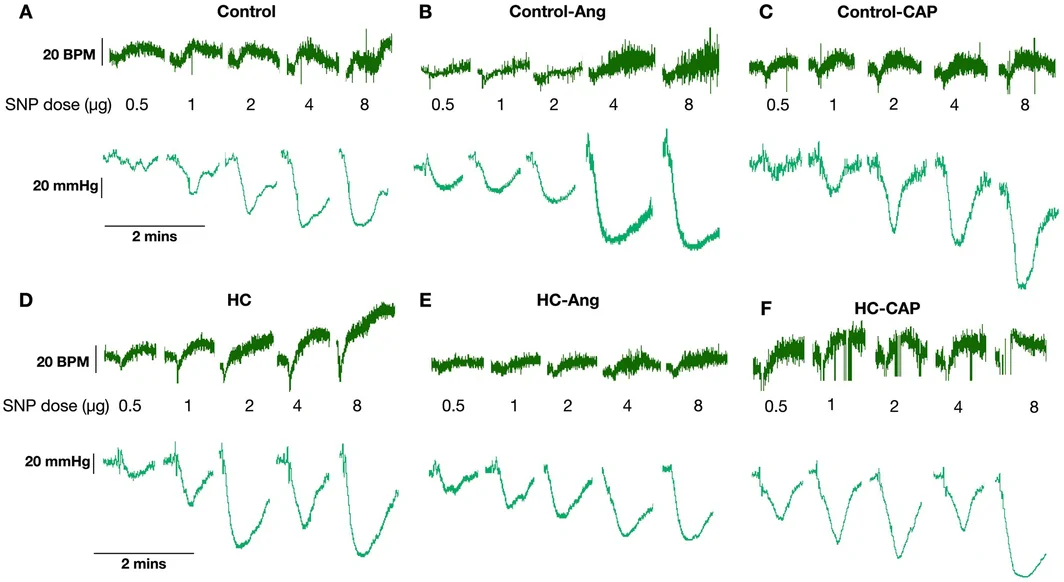
Representative tracings of sympathetic responses to sodium nitroprusside. Reflex tachycardic responses to blood pressure drops at increasing doses of the vasodepressor, sodium nitroprusside (SNP) in (A) Control, (B) Control‐Ang, (C) Control‐CAP, (D) HC, (E) HC‐Ang, and (F) HC‐CAP rats.

**FIGURE 7:**
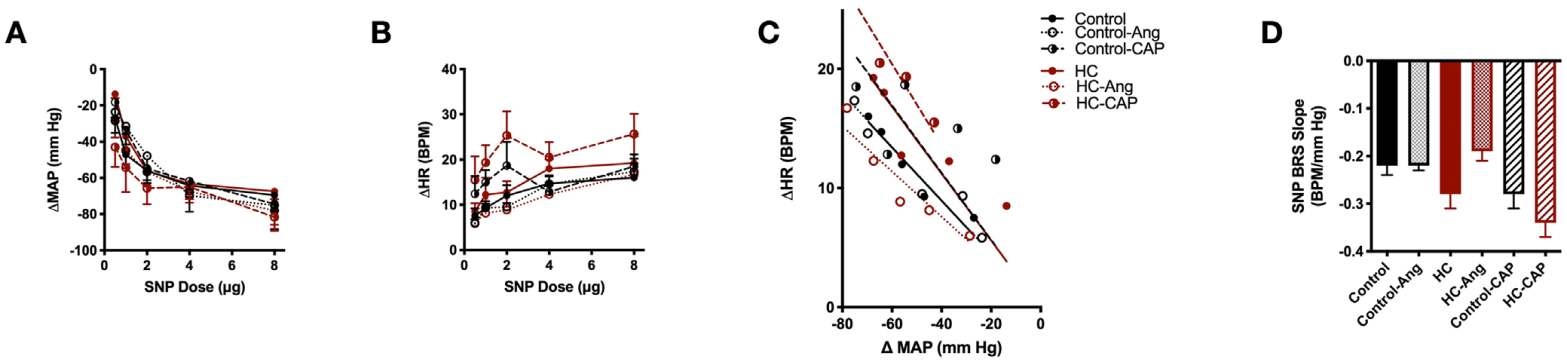
Sympathetic baroreceptor sensitivity assessed by invasive hemodynamic workup. (A) Changes in mean arterial pressure (ΔMAP, mmHg) and (B) Changes in heart rate (ΔHR, BPM) in response to increasing doses of sodium nitroprusside (SNP), (C) linear regression of ΔHR vs. ΔMAP, and (D) sympathetic baroreceptor sensitivity, as the slope of the regression line in the different rat groups. (Control: *n* = 8, HC: *n* = 8, Control‐Ang: *n* = 7, HC‐Ang: *n* = 8, Control‐CAP: *n* = 7, HC‐CAP: *n* = 7). Values are presented as mean ± SEM. Statistical significance was determined by one‐way ANOVA followed by Tukey's post hoc test for the comparison of baroreceptor sensitivity slopes (D) and two‐way repeated measures ANOVA followed by Sidak's post hoc test for the dose‐effect curves (A & B).

### 3.5 Cerebrovascular Tone

Consistent with previous results from our laboratory, middle cerebral arteriolar segments from HC‐fed rats (with saline pumps) exhibited significantly higher active tone compared to their control counterparts, specifically at 40 and 60 mmHg (*η*_*p*_^2^ group × pressure = 0.15) (Figure 8) [37]. Similarly, middle cerebral arterioles from HC‐fed rats with AngII infusion pump implants exhibited significantly higher active tone compared to their control counterparts (with AngII infusion pumps) at greater pressure steps, 120 and 140 mmHg (*η*_*p*_^2^ group × pressure = 0.51). On the other hand, no significant difference was observed in the active tone of middle cerebral arterioles of normal chow‐fed rats with AngII pumps compared to those with saline pumps or of HC‐fed rats with AngII pumps with respect to those with saline pumps, indicating that AngII infusion at the utilised dose and rate had no effect on active tone and that metabolic insult secondary to HC feeding conduced in exaggerated myogenic tone.

**FIGURE 8:**
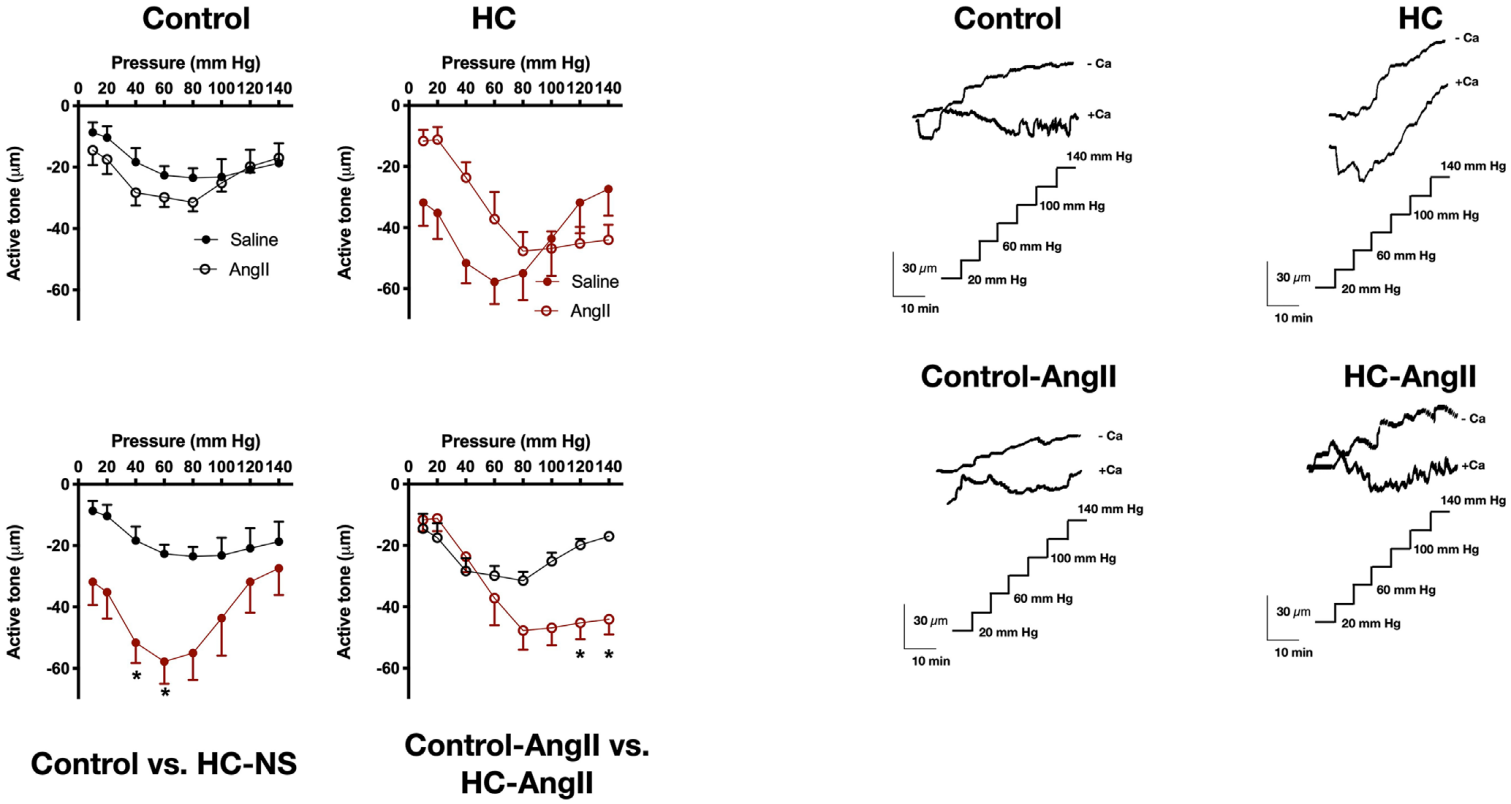
Examination of vascular active tone (μm) in middle cerebral arterioles by ex vivo pressure myography in Control (*n* = 5), HC (*n* = 5), Control‐Ang (*n* = 4), and HC‐Ang (*n* = 5) rats. Active tone was calculated as the difference of vascular diameters in calcium‐containing and calcium‐free buffer at the corresponding pressure points. Representative tracings are shown on the right. Values are presented as mean ± SEM. Statistical significance was determined by two‐way repeated measures ANOVA followed by Sidak's post hoc test for the different comparisons in each panel. * denotes *p* < 0.05.

### 3.6 Renovascular Function and Renal Perfusion

Examination of renovascular function revealed deterioration of endothelial function in HC‐fed rats (with saline pumps) which is further compounded by AngII infusion (Figure 9). In control rats, renal arteries elicit dose–response vasodilation to increasing doses of carbachol. Injection of L‐NAME and diclofenac into the perfusion solution evokes a significant reduction in endothelium‐dependent vasodilation at all except the lowest dose of carbachol, as demonstrated by significantly lower change in kidney perfusion pressure (*η*_*p*_^2^ group × concentration = 0.45). In contrast, HC feeding compromised endothelium‐dependent vasodilation as there was no significant difference in the change in perfusion pressure in the presence of nitric oxide synthase and cyclooxygenase inhibitors in HC‐fed rats. On the other hand, control rats with AngII infusion pumps maintained residual renovascular endothelial function, showing only slight blunting in the vasodilatory responses to higher doses of carbachol. A significantly lower change in perfusion pressure at higher doses of carbachol was still observed with Ang II infusion in the absence of metabolic insult (*η*_*p*_^2^ group × concentration = 0.66). A total loss of endothelium‐dependent vasodilation was evident in HC‐fed rats with AngII infusion pumps. Increasing doses of carbachol evoked an unexpected increase in perfusion pressure (positive change in perfusion pressure). No significant difference in the change of perfusion pressure was seen upon the addition of L‐NAME and diclofenac. Treatment of HC‐fed rats with a non‐hypotensive dose of captopril restored endothelium‐dependent vasodilation in renal arteries (*η*_*p*_^2^ group × concentration = 0.78).

**FIGURE 9:**
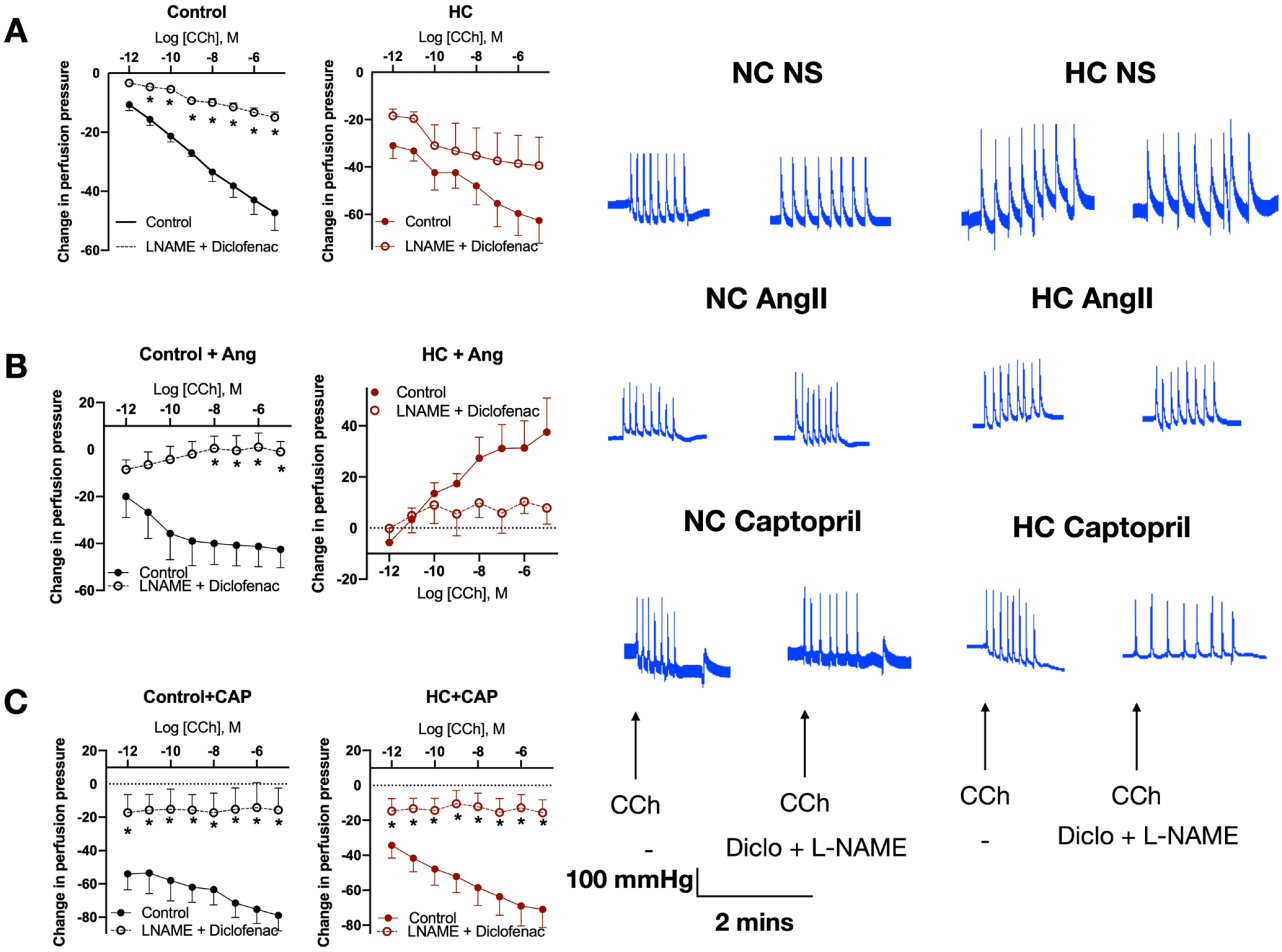
Examination of left renal artery endothelium‐dependent vasodilation. Change in perfusion pressure (mmHg) in response to increasing doses of carbachol (CCh, M), with and without L‐NAME and diclofenac in (A) Control and HC (*n* = 5 each), (B) Control + Ang (*n* = 4) and HC + Ang (*n* = 5), and (C) Control + CAP and HC + CAP rats (*n* = 4 each). Representative tracings are shown on the right. Values are presented as mean ± SEM. Statistical significance was determined by two‐way repeated measures ANOVA followed by Sidak's post hoc test for the different comparisons in each panel. * denotes *p* < 0.05.

### 3.7 PVAT and PRAT ACE Protein Concentration

The protein concentration of ACE in PVAT (Cohen's *d* = 1.59, 95% CI = 0.22–1.78) and PRAT (Cohen's *d* = 1.65, 95% CI = 0.29–1.47) of HC‐fed rats was significantly greater than that in their control counterparts (Figure 10).

**FIGURE 10:**
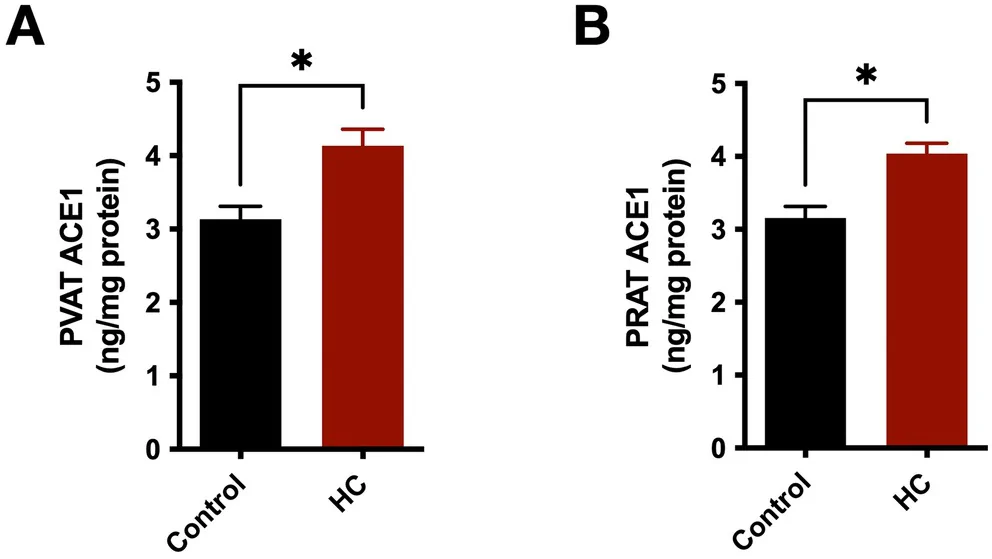
Quantification of protein concentration (ng/mg protein) of ACE1 in (A) PVAT and (B) PVAT of control and HC‐fed rats using ELISA. Values are presented as mean ± SEM. Statistical significance was determined by student *t*‐test for the different comparisons in each panel. * denotes *p* < 0.05.

### 3.8 Vascular Structure

In congruence with the absence of hypertension, examination of medial thickness using H&E staining reveals no significant difference among the different groups indicating the absence of hypertrophy in HC‐fed rats with saline or AngII infusion pumps (Figure 11A).

**FIGURE 11:**
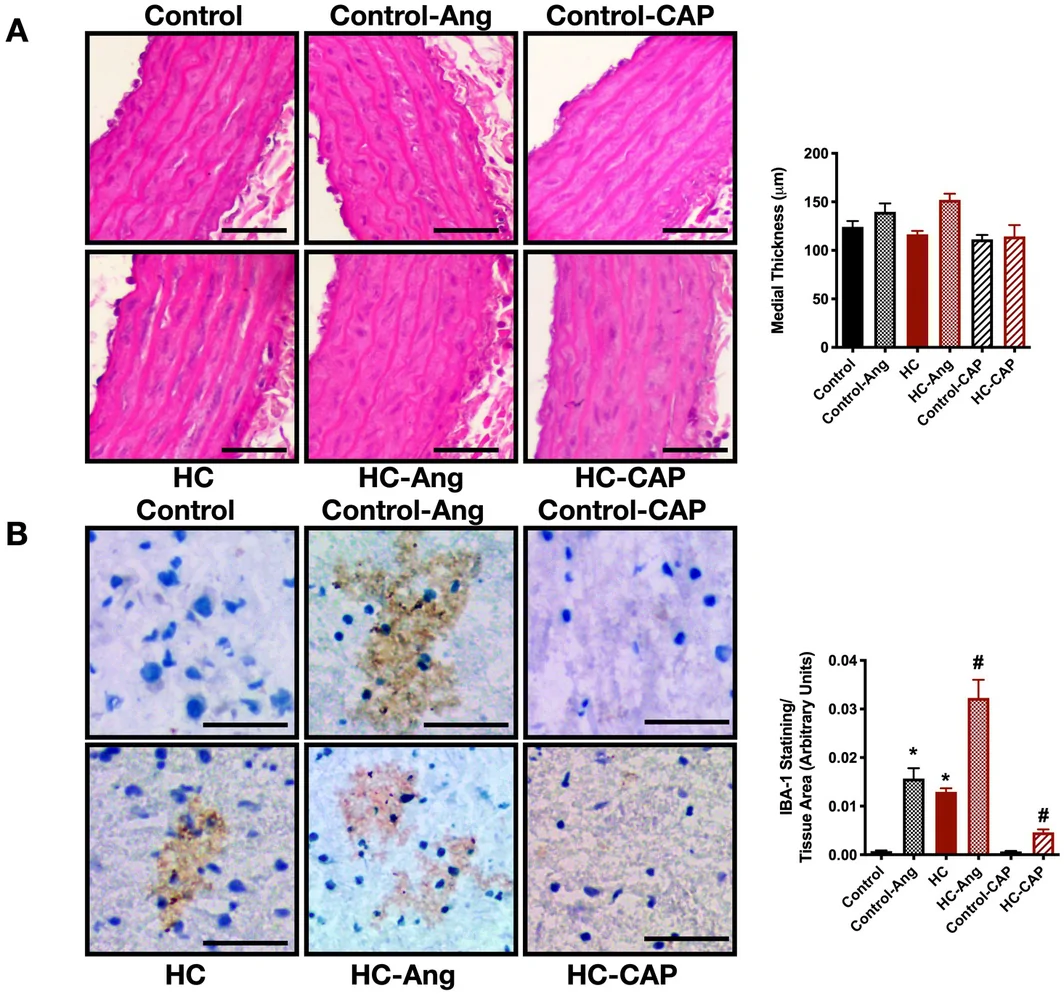
Examination of (A) thoracic aorta medial thickness using Haematoxylin and Eosin stain and (B) brainstem inflammation by immunological staining for IBA‐1. (*n* = 3/group) Representative micrographs are shown on the left. Scale represents 50 μm. Values are presented as mean ± SEM. Statistical significance was determined by one‐way ANOVA followed by Tukey's post hoc test. * and # denote *p* < 0.05 versus control and HC rats, respectively.

### 3.9 Brainstem Inflammation

Immunological staining for IBA‐1 showed a significant increase in brainstem inflammation in control rats with Ang II pumps and HC‐fed rats compared to control rats with saline pumps (Figure 11B). AngII infusion in HC‐fed rats aggravated brainstem inflammation, as demonstrated by significantly higher IBA‐1 stain compared to HC‐fed rats (with saline pumps). Treatment with captopril reduced brainstem inflammation. Brainstems of HC‐fed rats treated with captopril had significantly lower levels of IBA‐1 stain compared to their untreated counterparts.

### 3.10 Glomerular Retraction and Tubular Fibrosis

Control rats with AngII infusion as well as HC‐fed rats (with saline or AngII pumps) presented with significantly higher bowman capsule‐to‐glomerular area compared to control rats (normal chow‐fed with saline pumps), reflecting glomerular retraction (Figure 12). Treatment with captopril did not reverse glomerular retraction. HC‐CAP rats had significantly higher bowman capsule‐to‐glomerular area compared to control rats. On the other hand, Control‐AngII and HC‐fed rats had significantly higher tubular fibrosis compared to Control rats. Treatment with Captopril reduced tubular fibrosis of HC‐feeding (HC‐CAP).

**FIGURE 12:**
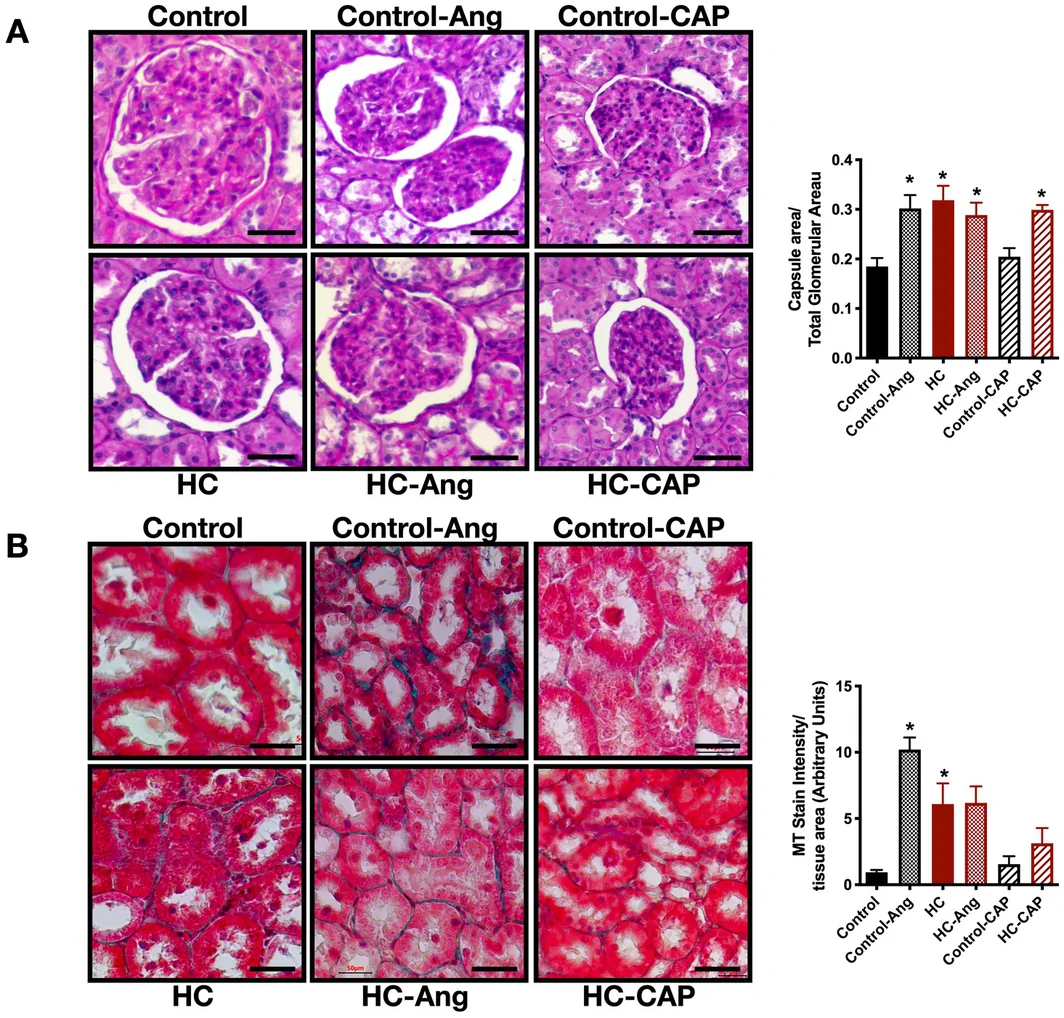
Assessment of (A) glomerular morphology and tubular fibrosis using Periodic Acid Schiff's and (B) Masson Trichrome stains, respectively (*n* = 3/group). Representative micrographs are shown on the left. Scale represents 50 μm. Values are presented as mean ± SEM. Statistical significance was determined by one‐way ANOVA followed by Tukey's post hoc test. * denotes *p* < 0.05.

## 4 Discussion

Diabetes is associated with an elevated morbidity and mortality profile related to cardiovascular, cerebrovascular, and renovascular complications [1, 2]. A significant body of evidence indicates that metabolic deterioration characteristic of the prediabetic state is at the origin of diabetic cardiorenal and cerebral disorders. We have previously demonstrated a role for PVAT and PRAT dysfunction in conducing cardiovascular and renovascular disorders and shown that activated thrombo‐inflammatory processes fan the flames of localised inflammation of the said adipose depots enveloping the vasculature [29, 31, 35, 37]. In this context, previous literature demonstrated the existence of RAS machinery in adipose tissues; however, molecular and functional characterisation of local RAS (over)activation in metabolic disease remains limited [38, 39]. A previous study reported a role for PVAT RAS overactivation in vascular dysfunction associated with heart failure and showed evidence of elevated ACE activity as well as AngII, AT1R, and AT2R expression in PVAT [40]. In mice, adipocyte angiotensinogen deficiency protects against obesity‐induced hypertension [41]. On the other hand, PVAT production of AngII has been shown to be limited by the uptake of liver‐produced angiotensinogen and blood‐derived renin [42].

In the present study, we characterise the role of RAS in subclinical cardiovascular and renovascular deterioration in a pre‐established normotensive rat model of prediabetes based on mild hypercaloric feeding. Our work on this model consistently showed cardioautonomic blunting, vascular hypercontractility, endothelial dysfunction, and a renovascular deficit leading to increased glomerular filtration and proteinuria [29, 30, 35, 43, 44]. As such, we started by establishing whether a framework exists for the involvement of aberrant ACE signalling in these disorders in the context of prediabetic metabolic dysfunction. Indeed, computational assessment demonstrated a significant probability for such an involvement given the extensive interaction among protein components of the RAS and those implicated in these disorders. This was particularly the case with insulin signalling that was common to the three examined disorders and was shown to be dysregulated in our rat model [29, 30, 37, 43, 44]. In such a framework, the protein interaction network emphasised a central role for ACE activity, and hence it would be expected that its modulation would have a potential ameliorative effect.

To validate the previous prediction, we treated prediabetic rats with a non‐hypotensive dose of the angiotensin‐converting enzyme inhibitor (ACEi), captopril. On the other hand, to isolate the effects of elevated systemic AngII levels, AngII infusion pumps were implanted. Hence, changes observed in prediabetic, saline‐infused rats were possibly attributable to autocrine and/or paracrine—but not endocrine—vascular effects of non‐hypertensive levels of AngII. From a different perspective, AngII infusion in prediabetic rats serves to assess the impact of further elevation in AngII levels—or that of superimposed hypertension—on premature prediabetic cardiovascular, renovascular, and cerebrovascular dysfunction associated with prediabetes. Moreover, comparison of the effects of AngII infusion on control versus HFD rats reveals the added deteriorative effect of the prediabetic state.

Our results reveal an activated RAS in prediabetes, despite the absence of obesity and hypertension, which contributes to reduced baroreceptor sensitivity of the parasympathetic arm and compromised renovascular endothelium‐dependent vasodilation. The latter is associated with tubular fibrosis which is responsive to captopril treatment. Previous studies have reported local, adipose‐produced components of the RAS [45]. Herein, we show a significant increase in ACE1 protein concentration in PVAT and PRAT of prediabetic rats compared to controls. Nevertheless, it is possible that prediabetes, per se, stimulates adipose as well as non‐adipose vascular AngII production.

Despite basic depot‐specific differences in RAS expression profiles, a previous study indicated that an obesogenic (high fat, high sucrose) diet activates adipose tissue classical RAS while a ketogenic diet upregulates the counterregulatory RAS arm in mice [46]. Intriguingly, RAS activation was demonstrated as elevated AT1R, while a reduction in white adipose tissues ACE1 expression was observed in response to both diets [46]. However, the assessment was limited to inguinal and epididymal white‐ and interscapular brown adipose tissues. We have previously shown that PVAT and PRAT exhibit elevated uncoupling protein 1 expression after 12 weeks of HC feeding, rendering them beige, possibly explaining differential ACE1 expression [30]. Importantly, adipose AngII production is under the effect of sympathetic stimulation [47, 48]. It has also been reported that insulin upregulates AngII production in adipose tissues of obese individuals in an inflammation‐mediated mechanism [27]. We have previously shown that our prediabetic rat exhibits hyperinsulinemia‐associated adipose tissue inflammation and that sympathovagal imbalance contributes to prediabetic PVAT and PRAT dysfunction‐driven cardio‐ and renovascular deterioration [34, 35, 37, 49]. As such, an upregulation of adipose RAS machinery in our prediabetic rat model is expected.

It is worth noting that adipose‐secreted ACE1 is responsible for extracellular conversion of AngI to AngII. Alternatively, adipocyte conversion of AngI to AngII is also carried out by the enzyme Chymase/Cathepsin G [50]. Thus, it can be speculated that the observed benefits of captopril may be related to the inhibition of adipose‐derived ACE driving the production of AngII in endothelial and/or vascular smooth muscle cells. Increased tissue AngII levels are associated with oxidative stress and reduced nitric oxide availability [51]. Our previous work revealed elevated levels of endothelial reactive oxygen species in the prediabetic rat, promoting changes in endothelial Kir channels which compromise endothelial nitric‐oxide‐dependent vasodilatory functions [34]. Moreover, ACE1 contributes to bradykinin breakdown. As such, restoration of bradykinin and downstream nitric oxide signalling secondary to the inhibition of augmented ACE1 activity could contribute to the observed ameliorative effects of captopril on renovascular function [52, 53, 54].

From an opposite point of view, the trophic, pro‐oxidative, and pro‐inflammatory effects of local, vascular AngII production on PVAT and PRAT cannot be precluded especially considering the expression of AT1 in adipose tissues [55, 56, 57, 58, 59, 60]. Indeed, activation of PVAT AT1R promotes adipose and vascular inflammation precipitating aneurysm [57]. Additionally, it has been demonstrated that ACE inhibition in mesenteric arteries reverses PVAT hypoxic milieu and restores its anticontractile capacity [61]. Hence, the amelioration derived from captopril could be the result of inhibiting vascular, that is, endothelial and/or smooth muscle cells, rather than adipose ACE activity and limiting the impact of vascular RAS activation on PVAT. Specifically, AngII is reported to inhibit adipogenesis, the result of which is enhanced hypertrophy of PVAT exposing the tissue to hypoxic and pro‐inflammatory stress [56]. Accordingly, we propose RAS overactivation in PVAT and PRAT as the result of and/or a contributor to adipose tissue and vascular inflammation in a vicious cycle, culminating in the observed early cardiac autonomic and renovascular deterioration.

Elevated systemic levels of AngII exacerbate prediabetic baroreceptor‐ and renovascular endothelial‐ but not cerebrovascular dysfunction, possibly indicating different pathological origins underlying prediabetic cardiorenal versus cerebrovascular manifestations. Indeed, cerebral vessels are devoid of PVAT. It has been previously shown that hypertension renders the blood–brain barrier permeable to AngII [62]. Our previous studies showed that this prediabetic rat model possesses an intact blood–brain barrier [37]. As such, the absence of an effect for systemic AngII on cerebrovascular dynamics indicates a preserved blood–brain barrier at the described level of serum AngII.

On the other hand, further deterioration of BRS and renovascular endothelial function in HC‐fed AngII‐infused rats compared to their saline‐infused counterparts supports the detrimental effect of systemic AngII on PVAT and PRAT exacerbating pre‐existing changes secondary to hyperinsulinemia [55, 56]. It is worth highlighting that hypertensive prediabetic rats exhibited significantly higher inflammation of the centre of cardioautonomic control, the brainstem, compared to their prediabetic only or normal‐chow fed, AngII‐infused counterparts and that captopril treatment reversed prediabetic brainstem inflammation. As captopril cannot access the blood‐brain barrier, the latter is likely a secondary effect of metabolic amelioration achieved with ACE inhibition [63].

A previous study revealed a causal effect for adipose tissue RAS on markers of systemic insulin sensitivity and intrarenal RAS activation [63]. Hence, our results are consistent with an upregulated RAS in PRAT of hyperinsulinemic rats, driving glomerular retraction and tubular fibrosis. In addition to paracrine control, adipose tissue afferent neurons relay central signals affecting renal and systemic functions [64].

Further molecular studies are needed to characterise the role of PVAT and PRAT RAS machinery in early prediabetic cardiorenal deterioration. Adipose tissue‐specific ACE1 knockdown can reveal the contribution of PVAT and PRAT RAS machinery to the observed vascular derangements. Moreover, ACE1 activity can be studied in primary co‐cultures of PVAT or PRAT and endothelial and/or vascular smooth muscle cells in the presence or absence of extracellular angiotensinogen to assess its contribution to AngII production. Additionally, to isolate the adipose‐tissue specific effects of captopril, it is necessary to investigate the status of the RAS machinery in vascular cells. As well, the absence of a group of prediabetic rats with AngII infusion pumps treated with captopril presents a possible limitation of our study. However, the purpose of the study was not to assess the effect of captopril treatment on cardiovascular, renovascular, and cerebrovascular manifestations of systemic AngII elevation but rather to characterise the contribution of subclinical, potentially local, RAS activation in the absence of hypertension, to early prediabetic deteriorative processes.

## 5 Conclusion

In this study, we present evidence of an activated, local RAS in our pre‐established rat model of normotensive prediabetes, contributing to early cardio‐ and renovascular deterioration, previously attributed to PVAT and PRAT dysfunction. We propose a role for elevated PVAT and PRAT ACE synthesis and release onto neighbouring vessels in exaggerated AngII‐related detrimental actions in endothelial cells that are reversed with ACE inhibition. Accordingly, targeted intervention provides a window for preventing and/or delaying diabetic cardiomorbidities that precedes the development of hypertension and its associated end‐organ damage. Moreover, intervening at the level of adipose tissue RAS with non‐hypotensive doses of an ACE inhibitor avoids the undesirable effects of systemic RAS manipulation and hemodynamic disruptions.

## Author Contributions

**Nour‐Mounira Z. Bakkar:** investigation, writing – original draft. **Rana Ghazi:** investigation. **Nahed Mougharbil:** investigation. **Safaa H. Hammoud:** investigation. **Ramzi Sabra:** writing – review and editing. **Ali H. Eid:** writing – review and editing. **Ahmed F. El‐Yazbi:** conceptualization, writing – original draft, resources, supervision, project administration, formal analysis, visualization, funding acquisition, writing – review and editing.

## Funding

The work was supported by the American University of Beirut, Faculty of Medicine, Medical Practice Plan (AUB‐MPP #320148) fund to Ahmed F. El‐Yazbi.

## Conflicts of Interest

The authors declare no conflicts of interest.

## Data Availability

Data are available from the corresponding author upon reasonable request.

## References

[1] N. D. Wong and N. Sattar , “Cardiovascular Risk in Diabetes Mellitus: Epidemiology, Assessment and Prevention,” Nature Reviews Cardiology 20 (2023): 685–695. 10.1038/s41569-023-00877-z 37193856

[2] E. W. Gregg , N. Sattar , and M. K. Ali , “The Changing Face of Diabetes Complications,” Lancet Diabetes & Endocrinology 4 (2016): 537–547. 10.1016/S2213-8587(16)30010-9 27156051

[3] J. M. Luther and N. J. Brown , “The Renin–Angiotensin–Aldosterone System and Glucose Homeostasis,” Trends in Pharmacological Sciences 32 (2011): 734–739. 10.1016/j.tips.2011.07.006 PMC3223326 21880378

[4] S. J. Forrester , G. W. Booz , C. D. Sigmund , et al., “Angiotensin II Signal Transduction: An Update on Mechanisms of Physiology and Pathophysiology,” Physiological Reviews 98 (2018): 1627–1738. 10.1152/physrev.00038.2017 PMC6335102 29873596

[5] E. S. Ford , G. Zhao , and C. Li , “Pre‐Diabetes and the Risk for Cardiovascular Disease: A Systematic Review of the Evidence,” Journal of the American College of Cardiology 55 (2010): 1310–1317. 10.1016/j.jacc.2009.10.060 20338491

[6] X. Cai , Y. Zhang , M. Li , et al., “Association Between Prediabetes and Risk of All Cause Mortality and Cardiovascular Disease: Updated Meta‐Analysis,” BMJ 370 (2020): m2297. 10.1136/bmj.m2297 PMC7362233 32669282

[7] H. Hu , T. Mizoue , N. Sasaki , et al., “Prediabetes and Cardiovascular Disease Risk: A Nested Case‐Control Study,” Atherosclerosis 278 (2018): 1–6. 10.1016/j.atherosclerosis.2018.09.004 30227266

[8] C. Welsh , P. Welsh , C. A. Celis‐Morales , et al., “Glycated Hemoglobin, Prediabetes, and the Links to Cardiovascular Disease: Data From UK Biobank,” Diabetes Care 43 (2020): 440–445. 10.2337/dc19-1683 31852727

[9] N. Stefan and M. B. Schulze , “Metabolic Health and Cardiometabolic Risk Clusters: Implications for Prediction, Prevention, and Treatment,” Lancet Diabetes & Endocrinology 11 (2023): 426–440. 10.1016/S2213-8587(23)00086-4 37156256

[10] A. K. Ziegler and C. Scheele , “Human Adipose Depots' Diverse Functions and Dysregulations During Cardiometabolic Disease,” NPJ Metabolic Health and Disease 2 (2024): 34. 10.1038/s44324-024-00036-z PMC11606922 39619657

[11] R. J. Garrison , W. B. Kannel , J. Stokes, III , and W. P. Castelli , “Incidence and Precursors of Hypertension in Young Adults: The Framingham Offspring Study,” Preventive Medicine 16 (1987): 235–251. 10.1016/0091-7435(87)90087-9 3588564

[12] W. B. Kannel , “Framingham Study Insights Into Hypertensive Risk of Cardiovascular Disease,” Hypertension Research 18 (1995): 181–196. 10.1291/hypres.18.181 7584927

[13] P. W. Wilson , R. B. D'Agostino , L. Sullivan , H. Parise , and W. B. Kannel , “Overweight and Obesity as Determinants of Cardiovascular Risk: The Framingham Experience,” Archives of Internal Medicine 162 (2002): 1867–1872. 10.1001/archinte.162.16.1867 12196085

[14] D. S. Hanes , A. Nahar , and M. R. Weir , “The Tissue Renin‐Angiotensin‐Aldosterone System in Diabetes Mellitus,” Current Hypertension Reports 6 (2004): 98–105. 10.1007/s11906-004-0083-8 15010011

[15] C. I. Johnston , “Tissue Angiotensin Converting Enzyme in Cardiac and Vascular Hypertrophy, Repair, and Remodeling,” Hypertension 23 (1994): 258–268. 10.1161/01.hyp.23.2.258 8307637

[16] M. Shargorodsky , E. Hass , M. Boaz , D. Gavish , and R. Zimlichman , “High Dose Treatment With Angiotensin II Receptor Blocker in Patients With Hypertension: Differential Effect of Tissue Protection Versus Blood Pressure Lowering,” Atherosclerosis 197 (2008): 303–310. 10.1016/j.atherosclerosis.2006.12.036 17588581

[17] I. AlZaim , A. H. Eid , K. S. Abd‐Elrahman , and A. F. El‐Yazbi , “Adipose Tissue Mitochondrial Dysfunction and Cardiometabolic Diseases: On the Search for Novel Molecular Targets,” Biochemical Pharmacology 206 (2022): 115337. 10.1016/j.bcp.2022.115337 36347272

[18] N.‐M. Z. Bakkar , I. AlZaim , and A. F. El‐Yazbi , “Depot‐Specific Adipose Tissue Modulation by SGLT2 Inhibitors and GLP1 Agonists Mediates Their Cardioprotective Effects in Metabolic Disease,” Clinical Science 136 (2022): 1631–1651. 10.1042/CS20220404 36383188

[19] N.‐M. Z. Bakkar , S. T. Osman , I. Alzaim , and A. F. El‐Yazbi , “Depot‐Biased ROS: A Middleman in Adipose‐Driven Cardiovascular Disease,” in Oxidative Stress in Cardiovascular‐Metabolic Diseases (Springer, 2024), 97–118.

[20] N. Alotaiq , A. S. Khalifa , A. Youssef , et al., “Targeting GSK‐3β for Adipose Dysfunction and Cardiovascular Complications of Metabolic Disease: An Entangled WNT/β‐Catenin Question,” FASEB Journal 38 (2024): e70273. 10.1096/fj.202402470R 39726401

[21] I. AlZaim , S. H. Hammoud , H. al‐Koussa , A. Ghazi , A. H. Eid , and A. F. el‐Yazbi , “Adipose Tissue Immunomodulation: A Novel Therapeutic Approach in Cardiovascular and Metabolic Diseases,” Frontiers in Cardiovascular Medicine 7 (2020): 602088. 10.3389/fcvm.2020.602088 PMC7705180 33282920

[22] S. H. Hammoud , I. AlZaim , Y. Al‐Dhaheri , A. H. Eid , and A. F. El‐Yazbi , “Perirenal Adipose Tissue Inflammation: Novel Insights Linking Metabolic Dysfunction to Renal Diseases,” Frontiers in Endocrinology 12 (2021): 707126. 10.3389/fendo.2021.707126 PMC8366229 34408726

[23] R. Rafeh , A. Viveiros , G. Y. Oudit , and A. F. El‐Yazbi , “Targeting Perivascular and Epicardial Adipose Tissue Inflammation: Therapeutic Opportunities for Cardiovascular Disease,” Clinical Science 134 (2020): 827–851. 10.1042/CS20190227 32271386

[24] A. F. El‐Yazbi , M. A. Elrewiny , H. M. Habib , A. H. Eid , P. A. Elzahhar , and A. S. Belal , “Thermogenic Modulation of Adipose Depots: A Perspective on Possible Therapeutic Intervention With Early Cardiorenal Complications of Metabolic Impairment,” Molecular Pharmacology 104 (2023): 187–194. 10.1124/molpharm.123.000704 37567782

[25] P. Balakumar , A. Alqahtani , N. A. Khan , T. Alqahtani , and G. Jagadeesh , “The Physiologic and Physiopathologic Roles of Perivascular Adipose Tissue and Its Interactions With Blood Vessels and the Renin‐Angiotensin System,” Pharmacological Research 173 (2021): 105890. 10.1016/j.phrs.2021.105890 34536547

[26] N. S. Kalupahana , F. Massiera , A. Quignard‐Boulange , et al., “Overproduction of Angiotensinogen From Adipose Tissue Induces Adipose Inflammation, Glucose Intolerance, and Insulin Resistance,” Obesity 20 (2012): 48–56. 10.1038/oby.2011.299 PMC4465436 21979391

[27] A. Harte , P. McTernan , R. Chetty , et al., “Insulin‐Mediated Upregulation of the Renin Angiotensin System in Human Subcutaneous Adipocytes Is Reduced by Rosiglitazone,” Circulation 111 (2005): 1954–1961. 10.1161/01.CIR.0000161954.17870.5D 15837949

[28] H. S. Dwaib and A. F. El‐Yazbi , “Protocol for Producing a Rat Model of Non‐Obese Prediabetes Using a Mild Hypercaloric Diet Approach,” STAR Protocols 5 (2024): 103276. 10.1016/j.xpro.2024.103276 PMC11387693 39178111

[29] O. Al‐Assi , R. Ghali , A. Mroueh , et al., “Cardiac Autonomic Neuropathy as a Result of Mild Hypercaloric Challenge in Absence of Signs of Diabetes: Modulation by Antidiabetic Drugs,” Oxidative Medicine and Cellular Longevity 2018 (2018): 9389784. 10.1155/2018/9389784 PMC5831709 29643979

[30] S. H. Hammoud , I. AlZaim , N. Mougharbil , et al., “Peri‐Renal Adipose Inflammation Contributes to Renal Dysfunction in a Non‐Obese Prediabetic Rat Model: Role of Anti‐Diabetic Drugs,” Biochemical Pharmacology 186 (2021): 114491, 10.1016/j.bcp.2021.114491. 33647265

[31] M. A. Elkhatib , A. Mroueh , R. W. Rafeh , et al., “Amelioration of Perivascular Adipose Inflammation Reverses Vascular Dysfunction in a Model of Nonobese Prediabetic Metabolic Challenge: Potential Role of Antidiabetic Drugs,” Translational Research 214 (2019): 121–143. 10.1016/j.trsl.2019.07.009 31408626

[32] H. S. Dwaib , G. Ajouz , I. AlZaim , et al., “Phosphorus Supplementation Mitigates Perivascular Adipose Inflammation–Induced Cardiovascular Consequences in Early Metabolic Impairment,” Journal of the American Heart Association 10 (2021): e023227. 10.1161/JAHA.121.023227 PMC9075232 34873915

[33] M. S. El‐Shoukrofy , A. Ismail , R. H. Elhamammy , et al., “Novel Thiazolones for the Simultaneous Modulation of PPARγ, COX‐2 and 15‐LOX to Address Metabolic Disease‐Associated Portal Inflammation,” European Journal of Medicinal Chemistry 289 (2025): 117415, 10.1016/j.ejmech.2025.117415. 40022874

[34] R. Alaaeddine , M. A. W. Elkhatib , A. Mroueh , et al., “Impaired Endothelium‐Dependent Hyperpolarization Underlies Endothelial Dysfunction During Early Metabolic Challenge: Increased ROS Generation and Possible Interference With NO Function,” Journal of Pharmacology and Experimental Therapeutics 371 (2019): 567–582. 10.1124/jpet.119.262048 31511364

[35] A. Al‐Saidi , I. F. Alzaim , S. H. Hammoud , et al., “Interruption of Perivascular and Perirenal Adipose Tissue Thromboinflammation Rescues Prediabetic Cardioautonomic and Renovascular Deterioration,” Clinical Science 138 (2024): 289–308. 10.1042/CS20231186 38381744

[36] N.‐M. Z. Bakkar , N. Mougharbil , A. Mroueh , et al., “Worsening Baroreflex Sensitivity on Progression to Type 2 Diabetes: Localized vs. Systemic Inflammation and Role of Antidiabetic Therapy,” American Journal of Physiology‐Endocrinology and Metabolism 319 (2020): E835–E851. 10.1152/ajpendo.00145.2020 32865011

[37] W. Fakih , A. Mroueh , H. Salah , et al., “Dysfunctional Cerebrovascular Tone Contributes to Cognitive Impairment in a Non‐Obese Rat Model of Prediabetic Challenge: Role of Suppression of Autophagy and Modulation by Anti‐Diabetic Drugs,” Biochemical Pharmacology 178 (2020): 114041, 10.1016/j.bcp.2020.114041. 32439335

[38] M. Pahlavani , N. S. Kalupahana , L. Ramalingam , and N. Moustaid‐Moussa , “Regulation and Functions of the Renin‐Angiotensin System in White and Brown Adipose Tissue,” Comprehensive Physiology 7 (2017): 1137–1150. 10.1002/cphy.c160031 PMC7561006 28915321

[39] P. Balakumar , K. M. Orayj , N. A. Khan , K. Shanmugam , and G. Jagadeesh , “Impact of the Local Renin–Angiotensin System in Perivascular Adipose Tissue on Vascular Health and Disease,” Cellular Signalling 124 (2024): 111461. 10.1016/j.cellsig.2024.111461 39389180

[40] M. T. Fontes , S. M. Paula , C. A. Lino , et al., “Renin–Angiotensin System Overactivation in Perivascular Adipose Tissue Contributes to Vascular Dysfunction in Heart Failure,” Clinical Science 134 (2020): 3195–3211. 10.1042/CS20201099 33215657

[41] F. Yiannikouris , M. Gupte , K. Putnam , et al., “Adipocyte Deficiency of Angiotensinogen Prevents Obesity‐Induced Hypertension in Male Mice,” Hypertension 60 (2012): 1524–1530. 10.1161/HYPERTENSIONAHA.112.192690 PMC3517298 23108647

[42] E. O. Cruz‐López , E. Uijl , and A. J. Danser , “Perivascular Adipose Tissue in Vascular Function: Does Locally Synthesized Angiotensinogen Play a Role?,” Journal of Cardiovascular Pharmacology 78 (2021): S53–S62. 10.1097/FJC.0000000000001027 34840262

[43] M. A. W. Elkhatib , A. Mroueh , R. W. Rafeh , et al., “Amelioration of Perivascular Adipose Inflammation Reverses Vascular Dysfunction in a Model of Non‐Obese Prediabetic Metabolic Challenge: Potential Role of Anti‐Diabetic Drugs,” Translational Research 214 (2019): 121–143, 10.1016/j.trsl.2019.07.009. 31408626

[44] R. Alaaeddine , M. E.‐K , A. Mroueh , et al., “Impaired Endothelium‐Dependent Hyperpolarization Underlies Endothelial Dysfunction During Early Metabolic Challenge: Increased ROS Generation and Possible Interference With NO Function,” Journal of Pharmacology and Experimental Therapeutics 371 (2019): 561–582. 10.1124/jpet.119.262048 31511364

[45] S. Engeli , R. Negrel , and A. M. Sharma , “Physiology and Pathophysiology of the Adipose Tissue Renin‐Angiotensin System,” Hypertension 35 (2000): 1270–1277. 10.1161/01.hyp.35.6.1270 10856276

[46] D. Da Eira , S. Jani , M. Stefanovic , and R. B. Ceddia , “Obesogenic Versus Ketogenic Diets in the Regulation of the Renin–Angiotensin System in Rat White and Brown Adipose Tissues,” Nutrition 105 (2023): 111862. 10.1016/j.nut.2022.111862 36356378

[47] E. E. Soltis and L. A. Cassis , “Influence of Perivascular Adipose Tissue on Rat Aortic Smooth Muscle Responsiveness,” Clinical and Experimental Hypertension. Part A, Theory and Practice 13 (1991): 277–296. 10.3109/10641969109042063 2065467

[48] C. Lu , L.‐Y. Su , R. M. Lee , and Y.‐J. Gao , “Mechanisms for Perivascular Adipose Tissue‐Mediated Potentiation of Vascular Contraction to Perivascular Neuronal Stimulation: The Role of Adipocyte‐Derived Angiotensin II,” European Journal of Pharmacology 634 (2010): 107–112. 10.1016/j.ejphar.2010.02.006 20156432

[49] A. F. El‐Yazbi , S. Hammoud , I. F. Alzaim , A. F. Bekdash , N. Mougharbil , and H. F. Itani , “Amelioration of Cardiac Autonomic Dysfunction Mitigates Perivascular and Perirenal Adipose Inflammation and Cardiorenal Involvement in Prediabetes,” FASEB Journal 36 (2022): R3343.

[50] B. Gálvez‐Prieto , J. Bolbrinker , P. Stucchi , et al., “Comparative Expression Analysis of the Renin‐Angiotensin System Components Between White and Brown Perivascular Adipose Tissue,” Journal of Endocrinology 197 (2008): 55–64. 10.1677/JOE-07-0284 18372232

[51] K. Chalupsky and H. Cai , “Endothelial Dihydrofolate Reductase: Critical for Nitric Oxide Bioavailability and Role in Angiotensin II Uncoupling of Endothelial Nitric Oxide Synthase,” Proceedings of the National Academy of Sciences 102 (2005): 9056–9061. 10.1073/pnas.0409594102 PMC1157015 15941833

[52] B. Hornig , C. Kohler , and H. Drexler , “Role of Bradykinin in Mediating Vascular Effects of Angiotensin‐Converting Enzyme Inhibitors in Humans,” Circulation 95 (1997): 1115–1118. 10.1161/01.cir.95.5.1115 9054837

[53] H. Oeseburg , D. Iusuf , P. van der Harst , W. H. van Gilst , R. H. Henning , and A. J. M. Roks , “Bradykinin Protects Against Oxidative Stress–Induced Endothelial Cell Senescence,” Hypertension 53 (2009): 417–422. 10.1161/HYPERTENSIONAHA.108.123729 19075096

[54] J. Lu , Q. Wang , W. Zheng , et al., “Captopril Alleviates Vascular Endothelial Dysfunction by Regulating TRPV1 and Activating the eNOS Pathway,” European Journal of Pharmacology 1031 (2026): 179172. 10.1016/j.ejphar.2026.179172 42537735

[55] Z. Wang , H. Lu , M. Garcia‐Barrio , et al., “RNA Sequencing Reveals Perivascular Adipose Tissue Plasticity in Response to Angiotensin II,” Pharmacological Research 178 (2022): 106183. 10.1016/j.phrs.2022.106183 35306139

[56] C. J. Rendon , S. W. Watts , and G. A. Contreras , “PVAT Adipocyte: Energizing, Modulating, and Structuring Vascular Function During Normotensive and Hypertensive States,” American Journal of Physiology‐Heart and Circulatory Physiology 328 (2025): H1204–H1217. 10.1152/ajpheart.00093.2025 PMC12125700 40250838

[57] T. Sakaue , J. Suzuki , M. Hamaguchi , et al., “Perivascular Adipose Tissue Angiotensin II Type 1 Receptor Promotes Vascular Inflammation and Aneurysm Formation,” Hypertension 70 (2017): 780–789. 10.1161/HYPERTENSIONAHA.117.09512 28760942

[58] A. B. Proença , G. R. Medeiros , G. S. Reis , et al., “Adipose Tissue Plasticity Mediated by the Counterregulatory Axis of the Renin‐Angiotensin System: Role of Mas and MrgD Receptors,” Journal of Cellular Physiology 239 (2024): e31265. 10.1002/jcp.31265 38577921

[59] B. Alexandre‐Santos , D.'A. C. Magliano , I. G. Giori , et al., “Renin‐Angiotensin System Modulation Through Enalapril and/or Exercise Training Improves Visceral Adiposity in Obese Mice,” Life Sciences 291 (2022): 120269. 10.1016/j.lfs.2021.120269 34974075

[60] M. C. Muñoz , J. F. Giani , F. P. Dominici , D. Turyn , and J. E. Toblli , “Long‐Term Treatment With an Angiotensin II Receptor Blocker Decreases Adipocyte Size and Improves Insulin Signaling in Obese Zucker Rats,” Journal of Hypertension 27 (2009): 2409–2420. 10.1097/HJH.0b013e3283310e1b 19901849

[61] C. A. Rosei , S. B. Withers , L. Belcaid , C. de Ciuceis , D. Rizzoni , and A. M. Heagerty , “Blockade of the Renin–Angiotensin System in Small Arteries and Anticontractile Function of Perivascular Adipose Tissue,” Journal of Hypertension 33 (2015): 1039–1045. 10.1097/HJH.0000000000000506 25909701

[62] V. C. Biancardi , S. J. Son , S. Ahmadi , J. A. Filosa , and J. E. Stern , “Circulating Angiotensin II Gains Access to the Hypothalamus and Brain Stem During Hypertension via Breakdown of the Blood–Brain Barrier,” Hypertension 63 (2014): 572–579. 10.1161/HYPERTENSIONAHA.113.01743 PMC4080808 24343120

[63] S. Kim , M. Soltani‐Bejnood , A. Quignard‐Boulange , et al., “The Adipose Renin‐Angiotensin System Modulates Systemic Markers of Insulin Sensitivity and Activates the Intrarenal Renin‐Angiotensin System,” BioMed Research International 2006 (2006): 027012. 10.1155/JBB/2006/27012 PMC1698261 17489015

[64] N. Ahmed , C. Dalmasso , M. B. Turner , G. Arthur , C. Cincinelli , and A. S. Loria , “From Fat to Filter: The Effect of Adipose Tissue‐Derived Signals on Kidney Function,” Nature Reviews Nephrology 21 (2025): 1–18. 10.1038/s41581-025-00950-5 PMC13555723 40175570

